# Wrist Hemiarthroplasty for Complex Intraarticular Distal Radius Fracture in a Patient with Manifest Osteoporosis

**DOI:** 10.3390/life12040471

**Published:** 2022-03-23

**Authors:** Matthias Holzbauer, Leonard S. Bodell, Stefan M. Froschauer

**Affiliations:** 1 Department of Orthopedics and Traumatology, Kepler University Hospital, Krankenhausstrasse 9, 4020 Linz, Austria; matthias.holzbauer@a1.net; 2 Medical Faculty, Johannes Kepler University Linz, Altenbergerstr 69, 4040 Linz, Austria; 3 Department of Orthopedics, St. Joseph’s Hospital and Medical Center, 500 W Thomas Rd., Phoenix, AZ 85013, USA; leonard.bodell@gmail.com

**Keywords:** complex intraarticular fracture, distal radius fracture, lunate degeneration, osteoporotic fracture, proximal row carpectomy, wrist hemiarthroplasty

## Abstract

Distal radius fracture (DRF) is one of the most common fractures of the elderly. The higher the degree of joint surface destruction, and the more adverse factors are involved, the more challenging proper treatment becomes. In this regard, osteoporosis as underlying systemic disease, chondropathy or degeneration of adjacent wrist bones as well as incompliance significantly impair the success of the chosen primary therapy. Wrist hemiarthroplasty has already been reported as primary or secondary procedure for DRFs. In this case report, we present a patient with a severely comminuted DRF including posttraumatic degeneration of the lunate as well as manifest osteoporosis. Wrist hemiarthroplasty using the ReMotion radius component in combination with proximal row carpectomy was performed as secondary surgery. This procedure proved to be a viable treatment option in terms of achieving low pain levels, high range of motion values and stable osteointegration over a course of 6.5 follow-up years.

## 1. Introduction

Distal radius fractures (DRFs) are one of the most common types of fracture [1]. Epidemiologic investigations found that the incidence of DRFs underlies two peaks with varying trauma mechanism, and affecting different patient populations: First, DRFs in younger age groups commonly affect boys and men with good bone stock and are associated with a high-energy trauma [2,3]. Secondly, DRFs in woman occur progressively with age from the perimenopausal period onwards due to a low-energy trauma, i.e., a simple fall from a standing position, with osteopenia/osteoporosis as underlying cause [2,3,4].

Historically, conservative treatment including closed reduction and a consecutive short arm splint represented the standard therapy for DRFs. Since the 2000s, volar locking plate systems have become increasingly used to perform an open reduction and internal fixation procedure [5]. Since then, the literature has not provided a consensus on a proper treatment algorithm, especially for comminuted fractures. Although volar locking plates are commonly used for stabilizing comminuted intraarticular fractures in patients with impaired bone density, loss of reduction and screw penetration have been described as drawbacks [6]. Thus, conservative treatment is often discussed as a reliable method in elderly patients with low demand and a high perioperative complication profile, even though this treatment is prone to a residual fracture displacement [7,8]. However, the mentioned complication profiles of volar locking plates, as well as the ones of conservative treatment including unacceptable reduction with intraarticular malunion with step-off and gap formation are claimed to cause radiocarpal osteoarthritis (OA) and loss of function [9].

Recently, wrist hemiarthroplasty (WHA) using the SOPHIA implant (Biotech, Paris, France), the Unicompartmental isoelastic resurfacing prosthesis (Prosthelast, Argomedical, Cham, Switzerland), or the Cobra implant (Groupe Lépine, Lyon, France) has been reported as salvage option in complex DRFs, if both osteosynthesis and conservative treatment imply a high risk of failure [10,11,12]. WHA was previously applied in cases where the prosthesis can articulate with an intact proximal carpal row [10,11,12].

This case report presents a patient with a complex, severely comminuted DRF and subsequent lunate degeneration, who was diagnosed with manifest osteoporosis. As secondary surgery, this patient received WHA implantation using the ReMotion radius component in combination with proximal row carpectomy (PRC). The aim of this report is to show the clinical and radiographical outcomes of this novel treatment approach with a follow-up period of 6.5 years.

## 2. Case Presentation

We present a case of a 73-year-old female patient who presented at our institution with massive pain and a malalignment of her left antebrachium after a fall on her left arm. Clinically, the patient revealed a massive swelling of the adjacent soft tissue and a numbness of the ulnar side of the ring finger and the whole little finger.

**Figure 1:**
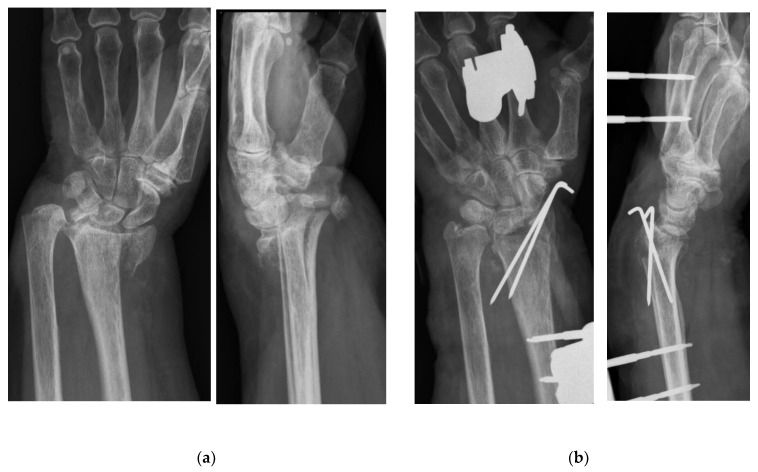
X-rays in posterior-anterior (left) and lateral (right) view after the initial patient’s presentation (**a**) as well as after K-wire and an external fixation installation (**b**).

X-rays followed by CT-imaging showed a comminuted fracture of the distal radius’ epi- and metaphysis with intraarticular involvement which was classified as Type C according to the Arbeitsgemeinschaft für Osteosynthesefragen/Orthopaedic Trauma Association (AO/OTA) system [13]. The radial as well as the ulnar styloid process were abrupted and dislocated (Figure 1a). Moreover, a bone fragment was compressing the ulnar nerve.

Due to massive swelling of the surrounding soft tissue, surgical treatment on the day of the injury included a closed reduction in the fracture and fixation of the large intraarticular fragments using 2 Kirschner-wires in plexus anesthesia. An external fixation, including two Schanz pins into the second metacarpal and into the radial diaphysis, was installed to add further stabilization on the complex fracture (Figure 1b). Moreover, an ulnar approach was used to remove the small bone adjacent to the ulnar nerve and to decompress the nerve; hence, the neurological complication of the fracture could be addressed.

During the hospital stay, a dual-energy X-ray absorptiometry (Discovery WI, Hologic Inc, USA) was used to measure bone mineral density: lumbar spine (L1–L4) and femoral neck revealed a T-score of −2.9 and −2.7, respectively. Thus, our patient could be diagnosed with manifest severe osteoporosis and the fracture was assessed as osteoporotic fracture. Consecutively, a proper osteoporosis treatment including an intravenous zoledronic acid infusion (5 mg/100 mL Aclasta^®^, Novartis Pharma Stein AG, Stein, Switzerland) was initialized. The preexisting vitamin D supplementation was increased to a sufficient level. Since then, the patient followed the annual bisphosphonate therapy and did not report to suffer from any side effects.

The neurological complications totally regressed within the first two postoperative weeks. In a CT-scan 3 weeks postoperatively; however, no bony union of the distal radius and a palmar dislocation of a fragment could be observed. Moreover, a dorsal dislocation of the distal ulna caused a total dislocation of the distal radioulnar joint. The external fixation was removed 4 weeks postoperatively and a forearm splint was applied, while the K-wires remained for two more weeks. Because of a lacking bone healing, an MRI-scan was conducted to screen the cartilage of the wrist joints: a chondropathy as well as a bone marrow oedema of the lunate (equivalent with Kienböck’s disease stage II) was detected. Moreover, we found a massive bone marrow edema and destruction of the joint surface of the distal radius.

Thus, we decided to perform a WHA as secondary surgery, which was finally performed 3 months after the initial trauma or first surgery, respectively. Our technique included a common dorsal approach and a PRC was performed as the first step [14]. Afterwards, the distal carpal row presented with an intact cartilage layer, while the joint surface of the distal radius was completely destroyed and palmarly dislocated. In accordance with the preoperative imaging, the fragments revealed no bony union. Furthermore, the distal radioulnar joint was dislocated leading to a relative ulna plus situation due to radius shortening. Thus, an ulna resection using the Darrach technique was performed. All mobile bone fragments of the distal radius were removed, and the remaining surface was flattened using an osteotome. The implantation of the ReMotion radius component (Stryker, Kalamazoo, MI, USA) was performed using the standard technique. The trial component in size M showed a stable fit and the capitate revealed a good articulation. Therefore, the original radius component in the same size was implanted without cement. Wound closure was conducted in a standard manner. Postoperative regimen included a thermoplastic splint for 4 weeks. Subsequently, the patient underwent intensive hand therapy for 6 months.

Postoperative follow-up examination revealed no signs for infection or implant loosening. Three weeks postoperatively, the patient presented with a non-inflamed olecranon bursitis, which could be successfully treated with anti-rheumatic drugs.

**Figure 2:**
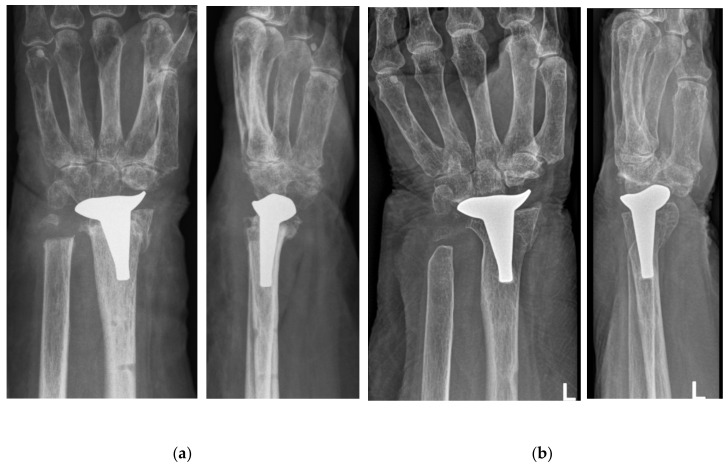
X-rays in posterior-anterior (left) and lateral (right) view 1 day after WHA (**a**). The follow-up X-ray after 6.5 years showed no sign of implant loosening, subsidence of carpal collapse (**b**).

A detailed follow-up examination was performed 1.5 years and 6.5 years postoperatively, including the Disability of the Arm, Shoulder and Hand (DASH) Score, pain evaluation via a visual analogue scale (VAS), range of motion measurement and radiographic assessments, as proposed by Boeckstyns et al. [15].The angle between the radial component and the long axis of the radius (Implant-Radius Angle), and the distance between the tip of the radial implant and the radial styloid (Implant-Styloid Distance) were measured. Postoperative as well as final radiographs after 6.5 years are displayed in Figure 2. We could not detect any sign of implant loosening, radiolucency, adjacent joint OA or collapse of the distal carpal row. The overall results are presented in Table 1. Moreover, the patient was subjectively content with the result at each follow-up appointment.

## 3. Discussion

This case report presents a patient who received implantation of a wrist hemiarthroplasty combined with PRC after suffering from a severely comminuted DRF. The clinical status prior WHA revealed a considerable, functional impairment as well as high pain scores, and poor range of motion values. This might be explained by the massively destructed distal radius joint surface, whose fragments could hardly be anatomically retained by means of initial surgery, i.e., external fixation and Kirschner wires. Arora et al. discovered in a large level I study including patients with DRF older than 65 years that there is hardly any difference in short-term outcomes between volar fixed-angle plate systems or cast immobilization [7]. In this regard, Herzberg et al. stated that primary anatomical reconstruction of “so-called irreparable DRF” is very difficult to achieve and secondary displacement is common [10,16]. Although elderly patients are often considered to have low demands, post-traumatically restricted range of motion and pain represent a high functional impairment of the respective upper extremity in independent elderly patients, as was the case in our patient [8,12]. Thus, WHA has previously been reported as viable option both in cases where reconstruction of the articular surface is initially difficult or impossible, and in DRFs with secondary displacement [10,12,16,17,18,19]. Various WHA systems have been used for these two indications: The SOPHIA prosthesis initially presented by Roux et al. in 2005 was one of the first ones for this indication. It is composed of a radial stem with a modular epiphyseal–metaphyseal block that articulates both with the carpus and the ulnar head [11,18]. Furthermore, the Cobra prosthesis first introduced by Herzberg in 2015 [10] and the Unicompartmental isoelastic resurfacing prosthesis [19] are more bone-sparing options. They do not require an intact distal ulna compared to the previous prosthesis [10]. Furthermore, Herzberg et al. included patients treated with the radial component of the ReMotion prosthesis system in two of their cases series [10,17]. The clinical outcomes of previous studies conducted on these four prostheses are well summarized by Benedikt et al. [12]. In general, the outcomes of the present case can be compared with the results presented in this narrative review [12].

Compared to the previously published studies presenting WHA techniques, our surgical method additionally included a PRC. The rationale behind this step was the posttraumatic chondropathy and advanced bone marrow oedema of the lunate. Thus, findings from idiopathic Kienböck’s disease proved that it is a progressive disease and surgical interventions should be considered from stage II onwards, which was the case in our patient [20]. Despite different etiologies, we decided in favor of a PRC because a rapid progression rate of 0.48 stages per year have been described for Kienböck’s disease [20]. Therefore, the proximal row was completely removed to prevent the patient from reoperations due to carpal OA. Although we could prove in a previous study that PRC is a reliable surgical technique for total wrist arthroplasty [14], we decided against implanting the carpal component of the ReMotion prosthesis due to the patient’s manifest osteoporosis. According to the World Health Organization, osteoporosis is defined as a metabolic bone disease characterized by low bone mass and microarchitectural deterioration of bone tissue causing progredient bone fragility and a higher risk for fracture [21]. We suspected that implanting the carpal component of the ReMotion prosthesis into osteoporotic bone stock would jeopardize stable osteointegration. Because the main force of the carpal prosthesis’ stem is transmitted into the capitate, which must be osteotomized at the proximal pole for implantation, we expected higher odds for subsidence or loosening in our patient. Moreover, periprosthetic osteolysis is a well-known, but insufficiently understood phenomenon in the postoperative course of total wrist arthroplasty, which might had further impaired prosthesis’ stability [15].

Thus, the biomechanical concept of our procedure is consistent with the one of a proximal row carpectomy; however, the intact capitate is articulating with the prosthesis instead of the lunate fossa of the radius [22]. Finally, our clinical outcomes can be compared with the ones of conventional PRC [22]. Despite the articulating surface being reduced to a low area, we could detect no radiographical complication in terms of adjacent joint OA or shortening of the capitate. In this regard, Herzberg stated that metal-on-cartilage contact is not the best contact for his WHA; however, this concept is already well established in shoulder and elbow salvage procedures [10,16].

Regarding osteoporosis, estimates have been made that there are currently 200 million people worldwide suffering from this condition [23]. Although isolated DRFs of elderly patients are not per se associated with an increased mortality [24], this fracture has emerged as indicator fracture for subsequent fractures of other sites, e.g., vertebrae and femoral neck; this phenomenon has previously been termed as “fracture cascade” [2,25]. These injuries are consecutively associated with high costs, morbidity, and mortality [2].

In the recent literature, bisphosphonate therapy showed a beneficial effect in prosthetic joint replacement, not only in patients with diagnosed osteoporosis; stress-shielding and implant wear with consecutive foreign body reaction are deemed to cause the well-known periprosthetic osteopenia [26]. Currently, meta-analyses showed that this periprosthetic bone loss could be significantly reduced by a bisphosphonate therapy, especially by third-generation drugs, both in the short and medium term [27,28]. The clinical relevance of this finding was controversially discussed until studies confirmed a beneficial effect on other end points: Ro et al. could prove a significantly reduced revision rate in patients receiving bisphosphonates after total knee and hip arthroplasty [29]. Considering these results, bisphosphonate therapy is of utmost importance in patients with diagnosed osteoporosis, not only for treating the underlying systemic disease and preventing subsequent fractures but also for maintaining stable osteointegration in case of the need for a prosthetic joint replacement. As it is the case in our patient, radiographic measurements proved that a stable prothesis’s fit could be achieved over the whole follow-up period.

The strength of the presents study is that the follow-up period exceeds the ones of previous studies covering WHA [12]. Thus, we can report the longest medium-term data showing continuously low pain levels and high range of motion values as well as the stable osteointegration. To the best of our knowledge, this study the first in the medical history reporting that PRC can be successfully added to a WHA procedure.

The limitation of the present case report is the experimental character of this procedure as a salvage procedure which will be reserved for a limited patient population. Moreover, there are no follow-up bone mineral density measurements available, which might also be considered as a limitation.

In conclusion, WHA in combination with PRC proved to be a new, viable treatment approach in a complex case of a severely comminuted DRF with posttraumatic degeneration of the lunate in a patient with manifest osteoporosis.

## Figures and Tables

**Table 1:** Functional parameters and radiographic measurements at baseline level and during follow-up examinations after WHA implantation.

Parameters	Preint.	1.5 Y	6.5 Y
DASH	86	5	38
VAS for Pain	7	1	0
Flexion	10°	40°	35°
Extension	0°	30°	35°
Radialduction	5°	10°	15°
Ulnarduction	15°	20°	20°
Pronation	30°	90°	90°
Supination	40°	90°	90°
Grip Strength	-	-	28 kg
Implant-Radius Angle	6°	8°	8°
Implant-Styloid Distance	35 mm	36 mm	37 mm

Preint. = preinterventionally.

## Data Availability

All data are included in the manuscript.
